# Supplementary material for: Maternal Dietary Zinc Intake during Pregnancy and Childhood Allergic Diseases up to Four Years: The Japan Environment and Children’s Study
Source: Nutrients. 2023 May 30;15(11):2568. doi: 10.3390/nu15112568 (PMC10255343; doi:10.3390/nu15112568)
Supplement: Supplementary file 1 [file nutrients-15-02568-s001.zip › nutrients-2395158-supplementary.pdf]

Supplementary Materials

Table S1 Baseline characteristics

Table S2 Characteristics of variables by whether used for analysis or not

Table S3 Odds ratios from logistic regression models with complete dataset

Table S4 Wald statistic for logistic models which contain a restricted cubic spline for adjusted maternal dietary zinc intake

Table S5 Wald statistic for logistic models which contain an interaction term between adjusted maternal dietary zinc intake and maternal history of allergy

Table S6 Logistic regression models further adjusted for paternal history of allergy

Table S7 Logistic regression models for subgroup, which excluded those who take vitamin or supplements during pregnancy

Table S1. Baseline characteristics.

**Variables**		**N**	**%**
Confounders			
Allergic disease history of mother	No	31068	41.7
	Yes	43496	58.3
History of abnormality of pregnancy	No	70052	93.9
	Yes	4512	6.1
	Missing	384	
Smoking status			
Mother	No	71401	96.4
	Yes	2690	3.6
	Missing	857	
Father	No	40781	55.7
	Yes	32450	44.3
	Missing	1717	
	Missing	384	
Low education status			
Mother	No	49482	66.7
	Yes	24656	33.3
	Missing	810	
Father	No	43102	58.4
	Yes	30676	41.6
	Missing	1170	
Low family Income	No	42985	61.6
	Yes	26798	38.4

	Missing	5165	
Feeding pet	No	57823	77.9
	Yes	16361	22.1
	Missing	764	
Pregnancy complications	No	62316	84.9
	Yes	11084	15.1
	Missing	1548	
Sex	Girls	36556	48.8
	Boys	38392	51.2
	Missing	0	
Pre-pregnancy overweight or Obesity	No	67502	90.1
	Yes	7397	9.9
	Missing	49	
Maternal age (years)	<35	55561	74.5
	>=35	19000	25.5
	Missing	387	
Maternal drinking	No	71927	97.3
	Yes	2010	2.7
	Missing	1011	
Parity	Nulliparous	30606	41.9
	Multipara	42514	58.1
	Missing	1828	
Low birth weight	No	68844	92.1
	Yes	5920	7.9
	Missing	184	
Premature birth	No	71448	95.5
	Yes	3358	4.5
	Missing	142	
Energy-adjusted zinc intake during pregnancy	Q3	15017	20.2
	Q1	14639	19.7
	Q2	14862	20
	Q4	15130	20.3
	Q5	14817	19.9
	Missing	483	
Ever wheeze (4y)	No	53220	71
	Yes	21728	29
	Missing	0	
Current wheeze (4y)	No	64309	85.8
	Yes	10639	14.2
	Missing	0	
Ever asthma (4y)	No	66104	88.2
	Yes	8844	11.8
	Missing	0	
AD (4y)	No	64797	86.5
	Yes	10151	13.5
	Missing	0	
Ever AD (4y)	No	65555	87.6

	Yes	9269	12.4
	Missing	124	
Ever rhinitis (4y)	No	48998	65.4
	Yes	25950	34.6
	Missing	0	
Rhinitis (4y)	No	51359	68.5
	Yes	23589	31.5
	Missing	0	
FA (4y)	No	70772	94.4
	Yes	4176	5.6
	Missing	0	
Current wheeze (3y)	No	59795	82.9
	Yes	12346	17.1
	Missing	2807	
AD (3y)	No	63370	87.8
	Yes	8822	12.2
	Missing	2756	
FA (3y)	No	68117	93.7
	Yes	4595	6.3
	Missing	2236	
Current wheeze (2y)	No	55155	76.2
	Yes	17199	23.8
	Missing	2594	
AD (2y)	No	63102	87
	Yes	9412	13
	Missing	2434	
FA (2y)	No	63972	88.6
	Yes	8221	11.4
	Missing	2755	
Current wheeze (2y)	No	59252	81
	Yes	13898	19
	Missing	1798	
AD (1y)	No	63472	86.7
	Yes	9733	13.3
	Missing	1743	
FA (1y)	No	68653	93.4
	Yes	4867	6.6
	Missing	1428	

AD: atopic dermatitis; FA: food allergy; BMI, body mass index.

Table S2. Characteristics of variables by whether used for analysis or not.

Variables		Not Used for Analysis		Data Used for Analysis	
		N	%	N	%
Allergic disease history of mother	Yes	14740	56.2	43496	58.3
History of abnormality of pregnancy	Yes	1743	6.6	4512	6.1
Smoking status					
Mother	Yes	2135	8.2	2690	3.6
Father	Yes	13829	54.5	32450	44.3
Low education status					
Mother	Low	11074	45.6	24656	33.3
Father	Low	12424	51.7	30676	41.6
Low family Income	Yes	10151	45.9	26798	38.4
Feeding pet	Yes	6333	26.1	16361	22.1
Pregnancy complications	Yes	4056	15.6	11084	15.1
Sex	Boys	13523	46.5	38392	51.2
Maternal BMI before pregnancy	>=25	3745	13.1	7397	9.9
Maternal age (years)	>=35	5674	21.7	19000	25.5
Maternal drinking	Yes	729	3.0	2010	2.7
Parity	Multipara	17631	63.2	42514	58.1
Low birth weight	Yes	4159	16	5920	7.9
Premature birth	Yes	3765	14	3358	4.5
Energy-adjusted zinc intake during pregnancy	Q1	5153	21	14639	19.7
	Q2	4940	20.1	14862	20
	Q3	4777	19.5	15017	20.2
	Q4	4670	19	15130	20.3
	Q5	4982	20.3	14817	19.9
Ever wheeze (4y)	Yes	1017	33.6	21728	29
Current wheeze (4y)	Yes	457	15.6	10639	14.2
Ever asthma (4y)	Yes	392	13.3	8844	11.8
AD (4y)	Yes	284	11.2	10151	13.5
Ever AD (4y)	Yes	443	14.2	9269	12.4
Ever rhinitis (4y)	Yes	1112	41.1	25950	34.6
Rhinitis (4y)	Yes	734	30.6	23589	31.5
FA (4y)	Yes	192	6.0	4176	5.6
Current wheeze (3y)	Yes	1880	19.6	12346	17.1
AD (3y)	Yes	1222	12.7	8822	12.2
FA (3y)	Yes	597	6.2	4595	6.3
Current wheeze (2y)	Yes	3311	25.5	17199	23.8

AD (2y)	Yes	1788	13.7	9412	13.0
FA (2y)	Yes	1629	13.1	8221	11.4
Current wheeze (2y)	Yes	3728	22.2	13898	19
AD (1y)	Yes	2249	13.4	9733	13.3
FA (1y)	Yes	1036	6.1	4867	6.6

AD: atopic dermatitis; FA: food allergy; BMI, body mass index.

Table S3. Odds ratios from logistic regression models with complete dataset.

			95% CI			95% CI	
		**OR** #	**Lower**	**Upper**	**OR** &	**Lower**	**Upper**
Ever wheeze	Q1	0.998	0.945	1.053	0.997	0.944	1.053
	Q2	0.978	0.926	1.032	0.977	0.925	1.031
	Q3	1.000			1.000		
	Q4	0.965	0.914	1.018	0.963	0.912	1.016
	Q5	0.975	0.923	1.029	0.972	0.920	1.026
Current wheeze	Q1	0.969	0.903	1.039	0.969	0.903	1.039
	Q2	0.959	0.894	1.028	0.958	0.893	1.027
	Q3	1.000			1.000		
	Q4	0.911	0.849	0.977	0.909	0.847	0.975
	Q5	0.988	0.921	1.060	0.985	0.918	1.057
Ever asthma	Q1	1.040	0.964	1.122	1.038	0.962	1.120
	Q2	0.973	0.901	1.050	0.973	0.901	1.050
	Q3	1.000			1.000		
	Q4	0.940	0.871	1.015	0.938	0.868	1.013
	Q5	1.036	0.960	1.118	1.032	0.956	1.114
Ever AD	Q1	1.060	0.985	1.141	1.060	0.985	1.142
	Q2	1.010	0.938	1.088	1.011	0.939	1.089
	Q3	1.000			1.000		
	Q4	0.942	0.874	1.015	0.943	0.875	1.016
	Q5	1.021	0.948	1.100	1.022	0.949	1.101
Current AD	Q1	1.031	0.960	1.107	1.031	0.960	1.107
	Q2	1.053	0.981	1.130	1.053	0.981	1.130
	Q3	1.000			1.000		
	Q4	0.971	0.904	1.042	0.971	0.905	1.043
	Q5	0.987	0.918	1.060	0.988	0.920	1.062
Ever rhinitis	Q1	1.025	0.974	1.080	1.025	0.973	1.079
	Q2	0.949	0.901	1.000	0.949	0.901	1.000
	Q3	1.000			1.000		
	Q4	0.961	0.913	1.012	0.961	0.913	1.012
	Q5	0.988	0.938	1.041	0.988	0.938	1.041
Current rhinitis	Q1	1.016	0.964	1.071	1.016	0.963	1.071

	Q2	0.955	0.905	1.007	0.955	0.905	1.007
	Q3	1.000			1.000		
	Q4	0.956	0.907	1.008	0.956	0.906	1.007
	Q5	0.976	0.925	1.029	0.976	0.926	1.030
Current FA	Q1	1.068	0.960	1.189	1.068	0.960	1.188
	Q2	1.015	0.911	1.130	1.014	0.911	1.129
	Q3	1.000			1.000		
	Q4	0.986	0.886	1.098	0.987	0.887	1.098
	Q5	1.056	0.950	1.175	1.057	0.950	1.176
Any allergy	Q1	1.022	0.972	1.074	1.021	0.972	1.073
	Q2	0.979	0.932	1.028	0.979	0.931	1.028
	Q3	1.000			1.000		
	Q4	0.941	0.896	0.988	0.940	0.895	0.988
	Q5	0.964	0.917	1.013	0.964	0.917	1.013

AD: Atopic dermatitis; FA: Food allergy; ORs: odds ratios; CI: confidence interval. # The models adjusted sex, parity, maternal age, pre-pregnancy overweight or Obesity, socioeconomic state variables, smoking status, maternal drinking, feeding pet, whether mother suffering allergic diseases before pregnancy and energy-adjusted zinc intake during pregnancy. & The models further adjusted low birth weight and premature birth.

Table S4. Wald statistic for logistic models which contain a restricted cubic spline for adjusted maternal dietary zinc intake.

Dependent Variables		Chi Square	df	P
Ever wheeze	Adjusted maternal dietary zinc intake	0.166	2	0.92
	Nonlinear	0.096	1	0.757
Current wheeze	Adjusted maternal dietary zinc intake	0.719	2	0.698
	Nonlinear	0.233	1	0.629
Ever asthma	Adjusted maternal dietary zinc intake	3.482	2	0.175
	Nonlinear	3.439	1	0.064
Ever AD	Adjusted maternal dietary zinc intake	1.859	2	0.395
	Nonlinear	1.439	1	0.23
Current AD	Adjusted maternal dietary zinc intake	6.351	2	0.042
	Nonlinear	1.765	1	0.184
Ever rhinitis	Adjusted maternal dietary zinc intake	7.114	2	0.029
	Nonlinear	5.395	1	0.02
Current rhinitis	Adjusted maternal dietary zinc intake	4.159	2	0.125
	Nonlinear	1.985	1	0.159
FA	Adjusted maternal dietary zinc intake	0.264	2	0.876
	Nonlinear	0.193	1	0.661
Any allergy	Adjusted maternal dietary zinc intake	5.599	2	0.061
	Nonlinear	0.029	1	0.865

AD: Atopic dermatitis; FA: Food allergy. The models also adjusted The models adjusted sex, parity, maternal age, pre-pregnancy overweight or Obesity, socioeconomic state variables, smoking status, maternal drinking, feeding pet, and whether mother suffering allergic diseases before pregnancy.

Table S5. Wald statistic for logistic models which contain a interaction term between adjusted maternal dietary zinc intake and maternal history of allergy.

Dependent Variables		Chi Square	df	P
Ever wheeze	Zinc	0.089	2	0.957
	Zinc × Maternal history of allergy	0.019	1	0.891
Current wheeze	Zinc	0.67	2	0.715
	Zinc × Maternal history of allergy	0.187	1	0.666
Ever asthma	Zinc	1.073	2	0.585
	Zinc × Maternal history of allergy	1.033	1	0.31
Ever AD	Zinc	1.494	2	0.474
	Zinc × Maternal history of allergy	1.083	1	0.298
Current AD	Zinc	4.726	2	0.094
	Zinc × Maternal history of allergy	0.051	1	0.822
Ever rhinitis	Zinc	5.066	2	0.079
	Zinc × Maternal history of allergy	3.363	1	0.067
Current rhinitis	Zinc	2.937	2	0.23
	Zinc × Maternal history of allergy	0.779	1	0.377
FA	Zinc	0.269	2	0.874
	Zinc × Maternal history of allergy	0.198	1	0.656
Any allergy	Zinc	5.886	2	0.053
	Zinc × Maternal history of allergy	0.316	1	0.574

AD: Atopic dermatitis; FA: Food allergy. The models also adjusted sex, parity, maternal age, pre-pregnancy overweight or Obesity, socioeconomic state variables, smoking status, maternal drinking, feeding pet.

Table S6. Logistic regression models further adjusted for paternal history of allergy.

			95% CI			95% CI	
		OR [#]	Lower	Upper	OR [&]	Lower	Upper
Ever wheeze	Q1	0.989	0.919	1.065	0.989	0.919	1.065
	Q2	0.981	0.912	1.056	0.981	0.911	1.055
	Q3						
	Q4	0.895	0.832	0.963	0.892	0.829	0.960
	Q5	0.935	0.869	1.007	0.933	0.867	1.005
Current wheeze	Q1	0.962	0.876	1.058	0.963	0.876	1.059
	Q2	0.976	0.889	1.072	0.976	0.889	1.072
	Q3						
	Q4	0.835	0.760	0.918	0.833	0.758	0.916
	Q5	0.972	0.885	1.068	0.970	0.884	1.066
Ever asthma	Q1	1.017	0.918	1.126	1.017	0.918	1.126

	Q2	0.960	0.866	1.063	0.960	0.866	1.063
	Q3						
	Q4	0.879	0.793	0.974	0.876	0.791	0.971
	Q5	0.983	0.888	1.089	0.982	0.886	1.087
Ever AD	Q1	0.988	0.892	1.095	0.989	0.892	1.096
	Q2	0.947	0.854	1.049	0.947	0.855	1.049
	Q3						
	Q4	0.910	0.822	1.008	0.912	0.824	1.010
	Q5	0.957	0.863	1.060	0.959	0.866	1.062
Current AD	Q1	0.980	0.888	1.081	0.979	0.887	1.080
	Q2	1.038	0.943	1.143	1.037	0.942	1.142
	Q3						
	Q4	0.995	0.904	1.094	0.996	0.905	1.095
	Q5	0.968	0.878	1.067	0.970	0.880	1.069
Ever rhinitis	Q1	1.017	0.947	1.092	1.017	0.947	1.092
	Q2	0.920	0.857	0.988	0.919	0.856	0.987
	Q3						
	Q4	0.983	0.917	1.054	0.983	0.917	1.054
	Q5	0.971	0.905	1.042	0.971	0.905	1.042
Current rhinitis	Q1	0.991	0.921	1.066	0.991	0.921	1.066
	Q2	0.931	0.866	1.001	0.930	0.865	1.000
	Q3						
	Q4	0.971	0.905	1.043	0.971	0.905	1.043
	Q5	0.952	0.886	1.024	0.952	0.886	1.024
Current FA	Q1	0.987	0.852	1.144	0.986	0.851	1.143
	Q2	1.008	0.872	1.165	1.007	0.871	1.163
	Q3						
	Q4	0.977	0.846	1.127	0.978	0.848	1.128
	Q5	0.992	0.859	1.146	0.993	0.859	1.147
Any allergy	Q1	1.014	0.947	1.085	1.013	0.947	1.085
	Q2	0.981	0.917	1.050	0.980	0.916	1.049
	Q3						
	Q4	0.961	0.899	1.027	0.961	0.899	1.027
	Q5	0.957	0.894	1.023	0.957	0.894	1.023

AD: Atopic dermatitis; FA: Food allergy; ORs: odds ratios; CI: confidence interval. [#] The models adjusted sex, parity, maternal age, pre-pregnancy overweight or Obesity, socio-economic state variables, smoking status, maternal drinking, feeding pet, whether mother suffering allergic diseases before pregnancy, allergic history of father and energy-adjusted zinc intake during pregnancy. [&] The models further adjusted low birth weight and premature birth.

Table S7. Logistic regression models for subgroup, which excluded those who take vitamin or supplements during pregnancy.

			95% CI			95% CI	
		**OR** #	**Lower**	**Upper**	**OR** &	**Lower**	**Upper**
Ever wheeze	Q1	0.974	0.906	1.048	0.974	0.905	1.047
	Q2	1.008	0.937	1.085	1.008	0.936	1.084
	Q3						
	Q4	0.981	0.911	1.057	0.980	0.910	1.055
	Q5	1.019	0.945	1.098	1.018	0.944	1.097
Current wheeze	Q1	0.981	0.893	1.077	0.980	0.893	1.077
	Q2	0.968	0.880	1.065	0.967	0.880	1.064
	Q3						
	Q4	0.907	0.823	0.999	0.904	0.821	0.996
	Q5	1.017	0.923	1.119	1.014	0.921	1.117
Ever asthma	Q1	1.046	0.944	1.158	1.044	0.943	1.155
	Q2	1.034	0.932	1.147	1.033	0.932	1.146
	Q3						
	Q4	0.983	0.884	1.092	0.980	0.882	1.088
	Q5	1.157	1.043	1.283	1.155	1.041	1.281
Ever AD	Q1	1.135	1.028	1.252	1.135	1.028	1.252
	Q2	1.027	0.927	1.136	1.027	0.928	1.137
	Q3						
	Q4	1.002	0.904	1.110	1.003	0.905	1.112
	Q5	1.032	0.930	1.145	1.033	0.931	1.146
Current AD	Q1	1.105	1.003	1.217	1.105	1.003	1.217
	Q2	1.099	0.997	1.212	1.100	0.998	1.213
	Q3						
	Q4	0.998	0.903	1.103	0.999	0.904	1.104
	Q5	1.027	0.928	1.136	1.028	0.929	1.137
Ever rhinitis	Q1	1.043	0.973	1.119	1.043	0.973	1.119
	Q2	0.980	0.913	1.052	0.980	0.913	1.052
	Q3						
	Q4	1.030	0.959	1.106	1.030	0.959	1.106
	Q5	1.056	0.983	1.135	1.057	0.983	1.136
Current rhinitis	Q1	1.035	0.963	1.112	1.035	0.964	1.112
	Q2	0.982	0.913	1.056	0.982	0.913	1.056
	Q3						
	Q4	1.025	0.953	1.103	1.025	0.953	1.102
	Q5	1.049	0.974	1.129	1.049	0.975	1.130
Current FA	Q1	1.076	0.932	1.243	1.076	0.932	1.243

	Q2	0.931	0.801	1.082	0.931	0.802	1.082
	Q3						
	Q4	0.919	0.790	1.068	0.920	0.791	1.070
	Q5	0.988	0.850	1.149	0.990	0.851	1.151
Any allergy	Q1	1.043	0.976	1.115	1.043	0.976	1.115
	Q2	1.012	0.946	1.082	1.012	0.946	1.082
	Q3						
	Q4	0.994	0.929	1.064	0.994	0.929	1.064
	Q5	1.010	0.943	1.082	1.010	0.943	1.082

AD: Atopic dermatitis; FA: Food allergy; ORs: odds ratios; CI: confidence interval. [#] The models adjusted sex, parity, maternal age, pre-pregnancy overweight or Obesity, socioeconomic state variables, smoking status, maternal drinking, feeding pet, whether mother suffering allergic diseases before pregnancy, allergic history of father and energy-adjusted zinc intake during pregnancy. [&] The models further adjusted low birth weight and premature birth.
